# Mental Health During the COVID-19 Pandemic in the United States: Online Survey

**DOI:** 10.2196/22043

**Published:** 2020-10-23

**Authors:** Jennifer S Jewell, Charlotte V Farewell, Courtney Welton-Mitchell, Angela Lee-Winn, Jessica Walls, Jenn A Leiferman

**Affiliations:** 1 Colorado School of Public Health Aurora, CO United States

**Keywords:** COVID-19, mental health, pandemic, depression, anxiety, well-being, stress

## Abstract

**Background:**

The COVID-19 pandemic has had numerous worldwide effects. In the United States, there have been 8.3 million cases and nearly 222,000 deaths as of October 21, 2020. Based on previous studies of mental health during outbreaks, the mental health of the population will be negatively affected in the aftermath of this pandemic. The long-term nature of this pandemic may lead to unforeseen mental health outcomes and/or unexpected relationships between demographic factors and mental health outcomes.

**Objective:**

This research focused on assessing the mental health status of adults in the United States during the early weeks of an unfolding pandemic.

**Methods:**

Data was collected from English-speaking adults from early April to early June 2020 using an online survey. The final convenience sample included 1083 US residents. The 71-item survey consisted of demographic questions, mental health and well-being measures, a coping mechanisms checklist, and questions about COVID-19–specific concerns. Hierarchical multivariable logistic regression was used to explore associations among demographic variables and mental health outcomes. Hierarchical linear regression was conducted to examine associations among demographic variables, COVID-19–specific concerns, and mental health and well-being outcomes.

**Results:**

Approximately 50% (536/1076) of the US sample was aged ≥45 years. Most of the sample was White (1013/1054, 96%), non-Hispanic (985/1058, 93%), and female (884/1073, 82%). Participants reported high rates of depression (295/1034, 29%), anxiety (342/1007, 34%), and stress (773/1058, 73%). Older individuals were less likely to report depressive symptomology (OR 0.78, *P*<.001) and anxiety symptomology (OR 0.72, *P*<.001); in addition, they had lower stress scores (–0.15 points, SE 0.01, *P*<.001) and increased well-being scores (1.86 points, SE 0.22, *P*<.001). Individuals who were no longer working due to COVID-19 were 2.25 times more likely to report symptoms of depression (*P*=.02), had a 0.51-point increase in stress (SE 0.17, *P*=.02), and a 3.9-point decrease in well-being scores (SE 1.49, *P*=.009) compared to individuals who were working remotely before and after COVID-19. Individuals who had partial or no insurance coverage were 2-3 times more likely to report depressive symptomology compared to individuals with full coverage (*P*=.02 and *P*=.01, respectively). Individuals who were on Medicare/Medicaid and individuals with no coverage were 1.97 and 4.48 times more likely to report moderate or severe anxiety, respectively (*P*=.03 and *P*=.01, respectively). Financial and food access concerns were significantly and positively related to depression, anxiety, and stress (all *P*<.05), and significantly negatively related to well-being (both *P*<.001). Economy, illness, and death concerns were significantly positively related to overall stress scores (all *P*<.05).

**Conclusions:**

Our findings suggest that many US residents are experiencing high stress, depressive, and anxiety symptomatology, especially those who are underinsured, uninsured, or unemployed. Longitudinal investigation of these variables is recommended. Health practitioners may provide opportunities to allay concerns or offer coping techniques to individuals in need of mental health care. These messages should be shared in person and through practice websites and social media.

## Introduction

The COVID-19 pandemic has produced over 41 million confirmed cases and over 1.1 million confirmed deaths worldwide as of October 21, 2020 [1]. Of these, nearly 8.3 million cases are in the United States, with nearly 222,000 deaths [1]. In addition to health impacts, many have raised the alarm about the potential for a widespread global mental health crisis as a result of the pandemic [2-5]. Specific groups may be at increased risk for adverse mental health outcomes, such as frontline health care workers [6] and those that have experienced illness or death of family, friends, or coworkers. Many more are likely to experience distress as a result of economic hardship, disruption to social networks, and work- and school-related changes due to the protracted crisis.

Elevated rates of depression and anxiety have been documented following stressors such as disease outbreaks, including the 2014-2016 Ebola crisis in West Africa, among caretakers, survivors, their immediate contacts, and others [7,8]. In addition, epidemics such as SARS and HIV have been associated with depression and other mental health concerns among various groups [9-14]. The current pandemic is likely to be associated with similar mental health outcomes, as a result of potential exposure to stressors including loss of loved ones, economic hardship, social isolation, and childcare responsibilities following school and day care closures.

Countless businesses across the United States closed in an attempt to protect workers, limit transmission of the coronavirus, and allow health care systems to keep pace with the needs of those requiring hospital care. With the exception of essential services, much of the economy has come to a virtual standstill, resulting in unprecedented rates of unemployment [15]. Financial struggles, including job loss and food insecurity, are known risk factors for mental illness, particularly anxiety, depression, and suicide [16,17].

In most US states, nonessential workers have been required to stay at home for several weeks. Many states have had stay-at-home orders in place for longer periods of time. Although there is an easing of movement restrictions in some areas within the United States, many people are still concerned about the potential safety risks of resuming prepandemic levels and types of activities. As a result, so-called “social distancing” continues for many in the United States. Physical distancing requirements (eg, social distancing) have the potential to limit physical and social contact, disrupt prepandemic social networks, and undermine the potential for social support at a time when it may be needed most. This may result in an increase in loneliness and social isolation. Across numerous studies, social isolation has been associated with increased morbidity and mortality, with an increase in coronary heart disease, stroke, and poor mental health outcomes such as depression and anxiety [18-22].

The increase in financial and familial struggles for some families may have exacerbated the negative effects of strict social distancing measures and overall trauma. Although studies examining the mental health impacts of COVID-19 are limited, findings from a few recent studies indicate that many in the United States are experiencing significant and worsening mental health difficulties during the pandemic [23]. A review of the emerging literature regarding the effects of the pandemic suggests that symptoms of anxiety and depression are common [24]. In one study [25], which used a representative sample and compared recent mental health concerns to those in 2018, large increases in mental health distress were noted. Younger people, those with children in the household, married individuals, and Asians appeared to be faring worse than others [25]. Authors suggested these findings may reflect economic hardship, but more research is needed to understand factors contributing to greater difficulties in some groups than others.

The current study examines demographic differences in mental health and well-being outcomes and specific sources of concern that impact these outcomes among a US sample of 1083 adults surveyed between April 7 and June 1, 2020, immediately following business closures and movement restrictions. This study may bring to light additional factors related to mental health during the pandemic and fill gaps in the current literature. Specifically, several COVID-19–specific concern-related items that have not been previously assessed were included in the current analyses. These findings have the potential to inform current intervention efforts as well as new initiatives, with the potential to mitigate suffering and bolster resilience during the ongoing pandemic.

## Methods

### Procedures

The Mental Health and Wellbeing Survey during COVID-19 Pandemic received ethical approval from the Colorado Multiple Institutional Review Board (COMIRB Protocol #20-0676). Survey data was collected between April 7 and June 1, 2020. A snowball sampling technique was used. This survey was advertised on Facebook and Instagram via paid targeted advertising. In addition, it was sent out via listservs and other media including Centers for Disease Control and Prevention (CDC) Prevention Research Centers, American Public Health Association Mental Health Section, Colorado Public Radio, University of Colorado research announcements, and the University of South Florida. Study data were collected and managed using REDCap electronic data capture tools hosted at the University of Colorado [26]. REDCap (Research Electronic Data Capture) is a secure, web-based application designed to support data capture for research studies, providing the following features: (1) an intuitive interface for validated data entry, (2) audit trails for tracking data manipulation and export procedures, (3) automated export procedures for seamless data downloads to common statistical packages, and (4) procedures for importing data from external sources. Participants consented digitally before beginning the survey. Additionally, participants in the initial survey were given the opportunity to opt in to future surveys to collect longitudinal data. A participation incentive in the form of a drawing for one of two $50 gift cards was offered.

### Participants

Adults aged ≥18 years were eligible to take the English-language survey, regardless of country of residence. There were no exclusion criteria beyond ability to provide consent. Although data was collected from an international sample initially, most of the participants were residing in the United States. As a result, only data from the US subsample is included in the present analyses. The final US sample consisted of 1083 individuals.

### Measures

The 71-item survey consisted of demographic questions, mental health and well-being measures, coping mechanisms, and questions gauging COVID-19–specific concerns. Demographic questions included age, race/ethnicity, gender, work status, household size, and insurance coverage. The survey also included four mental health and well-being scales measuring well-being, depression, anxiety, and stress. The Short Warwick-Edinburgh Mental Wellbeing Scale (SWEMWBS) was used as a continuous measure of well-being. It has high internal consistency and convergent validity with other measures of life satisfaction and physical and mental health (α=.93 in this sample). The SWEMWBS has a range of 7-35, with higher scores indicating higher well-being [27]. The Patient Health Questionnaire-2 (PHQ-2) was used as a brief measure of depression (α=.81 in this sample). The PHQ-2 has a sensitivity of 83% and a specificity of 92% for major depression. The PHQ-2 has a range of 0-6 and was dichotomized for analyses using a cutoff score of ≥3 [28,29]. Generalized Anxiety Disorder (GAD) was assessed using the GAD-7, which has a sensitivity of 89% and a specificity of 82% (α=.92 in this sample). The GAD-7 has a range of 0-21, and moderate or severe anxiety was based on a cut-off of ≥10 [30]. Lastly, stress was assessed using a validated 1-item continuous measure with 5 response options ranging from “not at all” to “very much” stress “these days” (Elo stress-symptoms item). This stress item has demonstrated construct, content, and criterion validity for group-level analysis [31].

The survey included a coping checklist, comprised of 12 behavioral items with an additional “other” option, to ascertain which types of coping were most common (eg, exercise, engaging with media, engaging remotely with family/friends). The survey items examining COVID-19–specific concerns included questions about personal financial impact, food security, economic impact, and risk of serious illness or death (in participants or others known to participants) related to COVID-19. Questions were phrased in the following manner: “How concerned are you about... [the financial impact current events may have on your family]?”

### Analyses

Data were exported from REDCap into SPSS (Version 25; IBM Corp) for analyses. Data cleaning included testing of assumptions, exploration of outliers, and missingness for all key variables. As all key variables had less than 10% missing data and data were missing completely at random (*χ^2^*_9_=12.86, *P*=.17), listwise deletion was used in all analyses. Univariate and bivariate analyses were conducted. Two proportion *z* tests were also used to calculate differences between responses (%) to the PHQ-2 and GAD-7 and national prevalence data. An independent sample *t* test was run to compare the sample average for the Warwick wellbeing score with a nationally representative sample.

Two hierarchical multivariable logistic regression models were run (logistic regression models 1 and 2) to explore associations among demographic variables, depression (not depressed versus depressed), and anxiety (no or mild anxiety versus moderate or severe anxiety) outcomes. Hierarchical regression was used to investigate if specific sources of concern (eg, financial concern, illness-related concern) were related to the outcome measures after controlling for demographic characteristics of the analytical sample. For categorical variables, well-established cutoffs based on representative US samples were used. All demographic variables were added simultaneously to each model, after which 5 unique sources of concern were entered into models (logistic regression models 3 and 4) to see which sources of concern predicted depression and anxiety outcomes after controlling for demographics. *R^2^* values, odds ratios, and *P* values for logistic regression models are presented.

Next, two hierarchical linear regression models were run (linear regression models 1 and 2) to explore associations between demographic variables and stress and well-being outcomes. In total, 5 unique sources of concern were entered into models (linear regression models 3 and 4) to see which sources of concern predicted stress and well-being outcomes after controlling for demographics. Unstandardized coefficients, *P* values, and adjusted *R^2^* values are reported for all linear regression models. Alpha (α) was set at .05.

## Results

Table 1 depicts demographics and the prevalence of mental health and well-being indicators of the final analytical sample, which included 1083 individuals. In total, 45 states within the United States were represented within the sample. Overall, 56% of the participants resided in Colorado. The remaining states comprised 0%-4% of the sample. Approximately 50% (536/1076) of the analytical sample were aged ≥45 years. Most of the sample was White (1013/1054,96%). Hispanic individuals made up 7% (73/1058) of the sample and 82% (884/1073) of participants were female. The average household size of the sample was 2.6 individuals. The self-reported depression rate in the sample population was 29% (295/1034) compared to a national average of 7% (*z*=27.8, *P*<.01) [32]. Approximately one-third of the sample reported moderate or severe anxiety (342/1007, 34%) compared to a national average of 20% of US adults prior to the pandemic (*z*=11.4, *P*<.01) [33]. Three-quarters (773/1058, 73%) of the sample reported experiencing stress “to some extent” or greater (“rather much” or “very much”). The average well-being score of the sample was 45.1 (SD 10.0), which compares to a national average of 51 (*t*[4349]=17.02, *P*<.01) [34]. From the 12 items provided (including the “other” specify option), the most prevalent coping behaviors reported by the sample included use of television (661/1083, 61%), texting with family and friends (661/1083, 61%), social media (617/1083, 57%), and exercise (617/1083, 57%).

Logistic regression models for the depression and anxiety outcomes are presented in Table 2. Age was related to mental health outcomes; older individuals were less likely to report depressive and anxiety symptomology compared to younger individuals (OR 0.78, 95% CI 0.70-0.87, *P*<.001 and OR 0.72, 95% CI 0.65-0.80, *P*<.001, respectively). Additionally, individuals who were no longer working due to COVID-19 were 2.25 times more likely to report symptoms of depression compared to individuals who were working remotely before and after COVID (“no change” group; 95% CI 1.15-4.43, *P*=.02). Finally, insurance status was significantly associated with both depression and anxiety outcomes. Individuals who had partial coverage and individuals with no coverage were 2.67 and 3.22 times more likely to report depressive symptomology compared to individuals with full coverage, respectively (95% CI 1.91-6.00, *P*=.02 and 95% CI 1.33-7.80, *P*=.01, respectively). Individuals who were on Medicare/Medicaid and individuals with no coverage were 1.97 and 4.48 times more likely to report moderate or severe anxiety compared to individuals with full coverage, respectively (95% CI 1.09-3.57, *P*=.03 and 95% CI 1.73-11.60, *P*=.01, respectively).

Linear regression models for the well-being and stress outcomes are presented in Table 3. An increase in age decade was associated with a 0.15-point decrease in stress score (SE 0.01, *P*<.001) and a 1.86-point increase in well-being score (SE 0.22, *P*<.001). On average, individuals who did not have insurance reported a 0.72-point higher stress score (SE 0.29, *P*=.002) and a 9.59-point lower well-being score (SE 2.09, *P*<.001). No longer working due to COVID-19 was associated with a 0.51-point increase in stress score and 3.90-point decrease in well-being score compared to individuals who were working remotely before and after COVID (“no change” group; SE 0.17, *P*=.02; SE 1.49, *P*=.009). Males also reported significantly lower stress scores compared to females (B=0.42, SE 0.10, *P*<.001).

Financial concerns and food access concerns were significantly and positively related to depression, anxiety, and stress (all *P*<.05) and significantly negatively related to well-being (both *P*<.001). Economy-, illness-, and death-related concerns were significantly and positively related to overall stress score after controlling for all demographic variables (all *P*<.05).

Additional analyses were considered, including investigating the effects of race/ethnicity and parenthood status. The cell sizes for these variables were too small to conduct analyses.

**Table 1:** Sample characteristics and mental health and well-being (N=1083).

Variables			Values
**Demographics**			
	**Age (years), n (%)**		
		18-25	46 (4.3)
		25-44	494 (45.9)
		45-59	313 (29.1)
		≥60	223 (20.7)
	**Race, n (%)**		
		White	1013 (96.1)
		Black or African American	4 (0.4)
		Asian	13 (1.2)
		Native Hawaiian or Pacific Islander	24 (2.3)
	**Ethnicity, n (%)**		
		Hispanic	73 (6.9)
		Non-Hispanic	985 (93.1)
	**Gender, n (%)**		
		Male	189 (17.6)
		Female	884 (82.4)
	**Health care insurance, n (%)**		
		Full coverage	893 (82.6)
		Partial coverage	38 (3.5)
		Medicaid/Medicare	120 (11.1)
		No insurance	30 (2.8)
	**Work status, n (%)**		
		Remote before and after COVID-19	104 (9.9)
		Unemployed prior to COVID-19	167 (15.9)
		Work outside home	120 (11.4)
		No longer working due to COVID-19	107 (10.2)
		Working remotely due to COVID-19	552 (11.4)
Household size, mean (SD)			2.6 (1.4)
**Mental health and well-being variables**			
	Warwick Wellbeing Scale, mean (SD)		45.1 (10.0)
	**Anxiety (GAD-7^a^), n (%)**		
		No/mild (<10)	665 (66.0)
		Moderate or severe (≥10)	342 (34.0)
	**Depression (PHQ-2^b^), n (%)**		
		Not depressed (<3)	739 (71.5)
		Depressed (≥3)	295 (28.5)
	**Stress, n (%)**		
		Not at all	67 (6.3)
		Only a little	218 (20.6)
		To some extent	363 (34.3)
		Rather much	240 (22.7)
		Very much	170 (16.1)

^a^GAD-7: Generalized Anxiety Disorder 7-item scale.

^b^PHQ-2: Patient Health Questionnaire 2-item scale.

**Table 2:** Logistic regression models showing associations between depression (Models 1 and 3), anxiety (Models 2 and 4), demographic variables, and sources of concern (N=1083).

Predictor variables		Model 1 (PHQ-2^a^)				Model 2 (GAD-7^b^)			Model 3 (PHQ-2)				Model 4 (GAD-7)			
		Odds ratio		*P* value		Odds ratio		*P* value	Odds ratio		*P* value		Odds ratio		*P* value	
Age		0.78		<.001^c^		0.72		<.001^c^	0.76		<.001^c^		0.68		<.001^c^	
**Race**																
	White		Ref^d^		Ref		Ref	Ref		Ref		Ref		Ref		Ref
	Black		2.13		.45		2.00	.49		0.75		.82		0.54		.64
	Native American or American Indian		1.91		.31		2.11	.24		1.98		.32		2.35		.21
	Asian		0.50		.24		0.41	.14		0.49		.25		0.34		.10
**Ethnicity**																
	Hispanic		Ref		Ref		Ref	Ref		Ref		Ref		Ref		Ref
	Non-Hispanic		1.34		.39		1.02	.94		1.43		.31		1.01		.99
**Work status**																
	Working remotely before and after COVID-19		Ref		Ref		Ref	Ref		Ref		Ref		Ref		Ref
	Unemployed prior to COVID-19		1.51		.22		0.92	.79		1.33		.42		0.69		.26
	Work outside home		1.00		.99		0.97	.93		1.00		.99		0.91		.78
	No longer working due to COVID-19		2.25		.02^e^		1.92	.06		1.80		.11		1.32		.45
	Working remotely due to COVID-19		0.75		.31		0.77	.36		0.72		.28		0.70		.22
**Insurance**																
	Full coverage		Ref		Ref		Ref	Ref		Ref		Ref		Ref		Ref
	Partial coverage		2.67		.02^e^		1.20	.67		2.35		.04^e^		1.05		.91
	Medicare/Medicaid		1.22		.49		1.97	.03^e^		1.19		.58		2.18		.02^e^
	None		3.22		.01^c^		4.48	<.001^c^		1.84		.20		3.09		.03^e^
**Gender**																
	Female		Ref		Ref		Ref	Ref		Ref		Ref		Ref		Ref
	Male		1.19		.41		0.75	.19		1.33		.22		0.91		.68
Household size		0.91		.12		0.97		.65	0.91		.13		0.99		.84	
Financial concern		N/A^f^		N/A		N/A		N/A	1.49		<.001^c^		1.32		.01^c^	
Food access concern		N/A		N/A		N/A		N/A	1.29		.01^c^		1.39		<.001^c^	
Economy-related concern		N/A		N/A		N/A		N/A	1.27		.08		1.29		.07	
Illness-related concern		N/A		N/A		N/A		N/A	0.97		.84		1.33		.12	
Death-related concern		N/A		N/A		N/A		N/A	1.21		.21		1.33		.07	
Adjusted *R*^2^		.07		N/A		.09		N/A	.13		N/A		.18		N/A	

^a^PHQ-2: Patient Health Questionnaire 2-item scale.

^b^GAD-7: Generalized Anxiety Disorder 7-item scale.

^c^*P*<.05.

^d^Ref: reference.

^e^*P*<.01.

^f^N/A: not applicable.

**Table 3:** Linear regression models showing associations between stress (Models 1 and 3), well-being (Models 2 and 4), demographic variables, and sources of concern (N=1083).

Predictor variables			Model 1 (stress)		Model 2 (well-being)		Model 3 (stress)		Model 4 (well-being)
			B (SE)		B (SE)		B (SE)		B (SE)
Age			–0.15 (.02)^a^		1.86 (0.22)^a^		–0.15 (0.02)^a^		1.91 (0.21)^a^
**Race**									
	White	Reference		Reference		Reference		Reference	
	Black	0.41 (0.54)		2.62 (4.71)		0.12 (0.55)		3.57 (5.23)	
	Native American or American Indian	0.36 (0.33)		2.18 (2.97)		0.33 (0.29)		2.30 (2.76)	
	Asian	–0.32 (0.25)		1.94 (2.24)		–0.32 (0.22)		2.15 (2.16)	
**Ethnicity**									
	Hispanic	Reference		Reference		Reference		Reference	
	Non-Hispanic	–0.08 (0.54)		2.62 (1.48)		–0.03 (0.15)		2.06 (1.44)	
**Work status**									
	Working remotely before and after COVID-19	Reference		Reference		Reference		Reference	
	Unemployed prior to COVID-19	–0.03 (0.16)		–2.81 (1.41)		–0.12 (0.14)		–2.09 (1.36)	
	Work outside home	0.04 (0.16)		0.03 (1.47)		0.02 (0.15)		0.12 (1.43)	
	No longer working due to COVID-19	0.51 (0.17)^a^		–3.90 (1.49)^a^		0.26 (0.15)		–2.58 (1.46)	
	Working remotely due to COVID-19	0.07 (0.13)		–0.33 (1.19)		0.05 (0.12)		–0.20 (1.16)	
**Insurance**									
	Full coverage	Reference		Reference		Reference		Reference	
	Partial coverage	0.50 (0.21)^b^		–2.20 (1.19)		0.37 (0.19)^b^		–1.57 (1.87)	
	Medicare/Medicaid	0.24 (0.14)		–1.77 (1.26)		0.21 (0.12)		–1.70 (1.22)	
	None	0.72 (0.29)^b^		–9.59 (2.09)^a^		0.28 (0.21)		–6.42 (2.06)^b^	
**Gender**									
	Female	Reference		Reference		Reference		Reference	
	Male	–0.42 (0.10)^a^		1.61 (0.91)		–0.28 (0.09)^b^		1.17 (0.89)	
Household size			–0.05 (0.03)		0.44 (0.25)		–0.04 (0.03)		0.50 (0.25)^b^
Financial concern			N/A^c^		N/A		0.28 (0.04)^a^		–1.41 (0.40)^a^
Food access concern			N/A		N/A		0.18 (0.04)^a^		–1.77 (0.42)^a^
Economy-related concern			N/A		N/A		0.15 (0.05)^b^		–0.79 (0.49)
Illness-related concern			N/A		N/A		0.15 (0.07)^b^		0.94 (0.69)
Death-related concern			N/A		N/A		0.13 (0.06)^b^		–1.37 (0.61)^b^
Intercept			4.21 (0.20)		33.94 (1.81)		1.79 (0.26)		43.63 (2.56)
Adjusted *R*^2^			.10		.12		.29		.19

^a^*P*<.05.

^b^*P*<.01.

^c^N/A: not applicable.

## Discussion

The imposed social distancing experienced by many throughout the United States undoubtedly contributed to numerous short- and long-term negative effects within the population. This survey aimed to identify the impact of the COVID-19 pandemic and imposed social distancing on mental health among US residents within a small window of time during which many businesses were closed and many individuals were out of work. Based on the findings associated with this convenience sample, when compared to prepandemic representative population-level data in the United States, it appears that mental health declined overall during the late spring of 2020. Prevalence rates of both depressive symptoms and anxiety symptoms were notably higher than national prepandemic averages. In addition, mental well-being significantly decreased, and stress levels were elevated in this sample. These findings support early evidence that the effects of the pandemic on mental health are significant [23].

The findings from the regression analyses suggest that age may be an important factor in considering mental health impacts of the pandemic. As age increased, anxiety symptoms, depression symptoms, and stress decreased, and well-being increased. This effect may be explained by stress on younger individuals due to inconsistent income or parenting-related obligations; however, these relationships could not be analyzed due to small cell sizes. Based on a review of the limited literature specifically related to the COVID-19 pandemic, Rajkumar [24] found that older adults were at greater risk for mental health concerns [35]. No other studies we reviewed found a relationship with age. Further research should be conducted to determine mental health risks relative to age and associated factors during the COVID-19 pandemic.

Findings from this study suggest loss of work due to pandemic-related closures greatly increased the odds of depression symptoms when compared to individuals who did not experience a change in their employment (were working remotely both before and after closures began). Loss of employment was also related to increased stress levels and decreased mental well-being. This could indicate a segment of the population that may require additional support to overcome mental health challenges during the pandemic. Economic crises have been tied to poor mental health outcomes in numerous studies [16,17]. Employment, in contrast to unemployment, has been linked to decreased mental illness, including depression and anxiety, and increased mental well-being [36]. Job instability, including moving from a permanent position to a temporary position, has been linked to increased mental illness [37]. Public health officials should make targeted efforts to reach out to the segment of the population that completely lost the ability to work during social distancing regulations. These individuals may need aid that extends beyond financial support.

Partial and no insurance coverage was associated with increased odds of depression symptoms when compared to fully insured individuals. This finding supports previous evidence that increased health care coverage reduces the prevalence of undiagnosed and untreated depression [38]. Individuals with limited health coverage also had higher stress scores and lower well-being scores. A similar effect was seen with moderate to severe anxiety. This finding was particularly pronounced in the uninsured population. The effects of partial or no insurance coverage on mental health may be exacerbated by the circumstances of the pandemic. Those with no insurance demonstrated extremely high odds of anxiety symptoms. This is likely related to concern about what would happen to them if they contracted COVID-19. Practitioners working with uninsured and partially insured individuals should take note of potentially decreased mental health in this population. Although these practitioners may not have the ability to affect their patients’ insurance status or concerns about the potential financial burden of contracting COVID-19, they do have the opportunity to encourage low- or no-cost coping methods that may decrease depressive and anxiety symptomatology.

Several other factors demonstrated relationships with mental health. Males reported significantly lower stress levels than females. This is consistent with findings on gender and stress [39]. This difference in stress levels may be due to gender differences in coping with stressful situations and differences in hormonal responses to stressful events [40]. Increased family financial concern and family food access concern were positively related with depression symptoms, anxiety symptoms, and stress, and negatively related to well-being. In addition, concern about the economy, illness-related concern, and death-related concern were positively related to stress scores. The financial concern and food security findings are consistent with previous work investigating this relationship [41,42]. Each of the relationships between the concern items and mental health variables is consistent with expected outcomes from the COVID-19 pandemic [43]. Practitioners may wish to ask their patients about specific concerns that they may be experiencing during this time. Using a sliding scale for medical fees and having referrals and information about different types of aid available (eg, food banks and local, state, and federal funds) may reduce the mental burden on some individuals. Practitioners are also in the best position to convey accurate information about COVID-19 risk status and effective protective measures. Information of this type can be conveyed in person or online through practice websites and social media. This reliable information may counteract the concern of illness and death and reduce poor mental health outcomes.

There are noteworthy limitations to this study. The convenience sample was primarily insured, non-Hispanic, White, and female, which may have led to results that are not generalizable to the broader population of US adults. Minority populations tend to experience the effects of trauma to a greater degree than others. Given the results seen in this study in a non-Hispanic White population that is primarily insured, it is reasonable to assume that minority populations may be impacted to an even greater degree than what was demonstrated in this study. Particular care should be taken to measure and address these concerns in future studies.

In addition, due to the small number of African Americans in this sample, we were not able to explore the relationship between race and mental health, a limitation that should be prioritized for exploration in follow-up research. In addition, the sample did not include a representative percentage of young people or individuals with children. Given the age effects in this study, further investigation is encouraged to determine the effect of age on mental health outcomes during the pandemic. The results of this study are based on a comparison with prepandemic norms, which may not be representative of the morbidity of these mental health conditions in peripandemic or postpandemic times. Functional impairment was not measured. Therefore, assumptions about the impact of negative mental health symptomatology in the peripandemic period cannot be made. Furthermore, the survey was conducted online, which likely inadvertently excluded individuals that do not have access to or are uncomfortable with the internet.

The strengths of this study include the large sample, which consisted of respondents from 45 of 50 states in the United States. This survey was also developed and launched early in the pandemic’s course through the United States. Therefore, it likely captured early mental health responses that later surveys may not have captured. These responses included both mental health struggles and positive mental health indicators. This study was designed with a follow-up in mind. Respondents to this survey were asked if they would be willing to participate in a follow-up survey at a later date. This will allow for longitudinal data collection at multiple time points as social distancing restrictions change throughout the United States.

Our findings suggest that many US citizens, particularly non-Hispanic, White, insured individuals, are experiencing high stress, depressive, and anxiety symptomatology. Practitioners, including health care workers and mental health specialists, can be a resource for those struggling with mental health concerns during the pandemic. These messages should not only be made in person, but also through practice websites and social media accounts. The overwhelming amount of information available to the public regarding COVID-19 makes it difficult to delineate accurate information from inaccurate information [44]. Practitioners have a preexisting rapport with their patients that they should use to shift the balance toward accurate information. This patient-provider relationship may engender trust that does not exist with larger health or government entities. Practitioners should capitalize on this rapport to convey accurate, timely information regarding risk factors, protective measures, coping techniques, financial relief, and food banks.

Policy makers should encourage growth in areas of mental health support that are most feasible during this time. Telemental health, for example, has been shown to be highly effective, cost-efficient, and accessible, especially in isolated communities [45]. Online mental health assessments and self-directed mental health interventions have also been widely introduced in China, with their effectiveness remaining to be seen [46].

Future research should continue to track the mental health effects of the pandemic as it progresses. There may be future waves of illness that impact social distancing recommendations and requirements. These, in turn, may impact mental health. Longitudinal investigation of these effects is recommended. Future studies should make concerted efforts to obtain a representative sample. Representative state-specific samples are available through various entities for a fee. In addition, specific outreach to underrepresented populations is recommended. Knowledge of these fluctuations in population mental health can be used by public health practitioners, mental health practitioners, and policy makers in their decision making and in their framing of recommendations.

## Abbreviations

GAD-7: Generalized Anxiety Disorder 7-item
PHQ-2: Patient Health Questionnaire-2
REDCap: Research Electronic Data Capture
SWEMWBS: Short Warwick-Edinburgh Mental Well-being Scale
